# Micro-mapping of terrestrial gamma radiation dose rate in typical urban homes in Miri City (Sarawak, Malaysia)

**DOI:** 10.1007/s10967-023-08838-z

**Published:** 2023-03-08

**Authors:** Dominique Dodge-Wan, Prasanna Mohan Viswanathan, Sheng Qin Seow

**Affiliations:** Department of Applied Sciences, Faculty of Engineering and Science, Curtin University Malaysia, CDT 250, 98009 Miri, Sarawak Malaysia

**Keywords:** Gamma, Dwellings, Building materials, Tiles, Annual exposure dose

## Abstract

Micro-mapping of terrestrial gamma radiation dose (TGRD) at meter grid spacing in and around four urban homes in Miri City shows rates ranging from 70 to 150 nGy/h. Tiled surfaces (floors and walls) vary between properties and have a clear and significant influence on TGRD which is highest in kitchens, washrooms and toilets. Application of a single indoor value for annual effective dose (AED) may lead to underestimations of up to 30%. The AED is unlikely to exceed 0.8 mSv in homes of this type in Miri, which is within recommended guidelines.

## Introduction

Background radiation from natural materials in our everyday environment, like air, soil, rocks and water, is one component of the exposure humans are subjected to, in addition to cosmic radiation [1]. Background radiation depends on a number of factors that are different from place to place, for example, the nature of the underlying geological material [2–4]. In addition, humans living in urban environments may also be subjected to external radiation emanating from various materials found in the built environment. Furthermore, radon gas concentrations may contribute to natural radioactivity and the total effective dose that populations are exposed to [5, 6] since inhalation of radon gas is a potential source of internal exposure. In general, absorbed dose rates in urban and built-up areas are expected to exceed those in rural areas of similar geology due to the effect of building materials.

Buildings may be constructed using a number of geological or geologically-derived materials, the source of which may be local, regional or international, and these source materials may contain variable amounts of radionuclides and their progenies. Among the common geological materials used in construction are clay (in bricks), limestones, clay and quartz sand (in cement), feldspars, granites, marbles and zircon used in glazing for tiles and ceramics as well as various rocks used for aggregate. Industry by-products such as coal fly ash, alum shale or phosphogypsum may also be incorporated into building materials with radiological implications [6, 7].

Other studies have shown that “choice in building materials has a noticeable contribution towards the indoor doses inhabitants are exposed to” [8] and in particular the zircon used in the tile gazing process [9]. In a study by Dodge-Wan and Mohan Viswanathan on Curtin University campus located in the north of Miri, tiles were found to contribute to gamma dose with an average indoor-to-outdoor TGRD ratio of 1.4 [10].

A number of models have been proposed for estimating the gamma dose indoors based on characteristics of the building materials and parameters related to the construction. Typical parameters used in these simulation models are room dimensions, wall thicknesses and density of floor, the surface of tiled areas, wall and ceiling materials, and the activity concentrations of ^226^Ra, ^232^Th and ^40^K of the substances used i.e. their composition [11]. The RESRAD-BUILD computer code is an example of such a model [8, 12–14]. This has led to numerous studies that have focused on measuring those activity concentrations in various building materials [15]. In Malaysia for example, Yasir and Yahaya [16] studied 13 types of building materials available locally, while more recently Abdullahi et al. [17] studied 102 types and Abdullahi et al. [13] studied 80 types.

Since the time spent indoors can account for 80% of a person’s life, it is important to accurately assess the indoor component of an annual effective dose. A growing number of studies worldwide have measured gamma dose indoors in situ, as opposed to calculating it based on other data [2, 5, 18–25].

Mollah et al. [18] used dosemeters and survey meters to measure environmental gamma radiation in 20 homes constructed out of natural materials, in villages near Cox’s Bazar, Bangladesh an area of high natural background radiation. Miah [19] measured indoor gamma dose rates for a period of a year in 15 brick and concrete buildings in Dhaka, Bangladesh. Al-Ghorabie [20] used dosemeters over a year to compare indoor gamma radiation in 250 houses in the city of At-Taif, Saudi Arabia including in apartments and mud houses, halls and villas with readings in one room per house. Malathi et al. [21] measured indoor gamma radiation but limited that to inside bedrooms in Coimbatore City, India and it is not reported how many readings were taken. Al-Saleh [22] used dosimeters over a 9-month study period to assess indoor gamma in various living rooms, bedrooms, kitchens and bathrooms in 5 homes in Riyadh city, Saudi Arabia. Svoukis and Tsertos [5] measured gamma radiation in situ in 70 locations outdoors and 20 indoors in urban areas in Cyprus and found an indoor-to-outdoor ratio of 1.4 ± 0.5. Papachristodoulou et al. [23] measured gamma radiation levels indoors and outdoors in 42 workplaces on a university campus in Greece. Hashemi et al. [24] measured gamma radiation in 43 randomly-selected homes in the city of Tehran, Iran but without mention of the type of building or rooms.

In general, these studies tend to be limited to a few isolated or single measurements in a large number of homes. An example is a study by Sakellariou et al. [26] in which 651 homes across 33 cities in Greece were monitored for indoor radiation. As a result, there is a research gap for detailed mapping of TGRD based on numerous in situ measurements within the different rooms and spaces inside typical homes i.e. micro-mapping of TGRD. Hence, this research aims to map the distribution of TGRD within typical urban homes in Miri City (Sarawak, Malaysia) and to assess how TGRD varies spatially and according to the specific building materials present. This study is based on a large number of in situ measurements from within four typical urban homes in Miri. The data can then be used to calculate the external exposure i.e. radiological impact of living in rooms with a range of construction materials within typical urban homes as well as the worst-case scenario of spending a large amount of time in those rooms with the highest radiological impact. This study does not cover internal exposure, which can be caused by inhaling ^222^Rn, a decay product of ^238^U and which is considered the most significant radionuclide that can accumulate in poorly ventilated dwellings and basements [22]. Radon in dwellings is generally tested using lithium fluoride thermoluminescence dosemeters which are passive monitors left in place over periods of 3 months or more [18, 21]

## Study area

**Fig.1:**
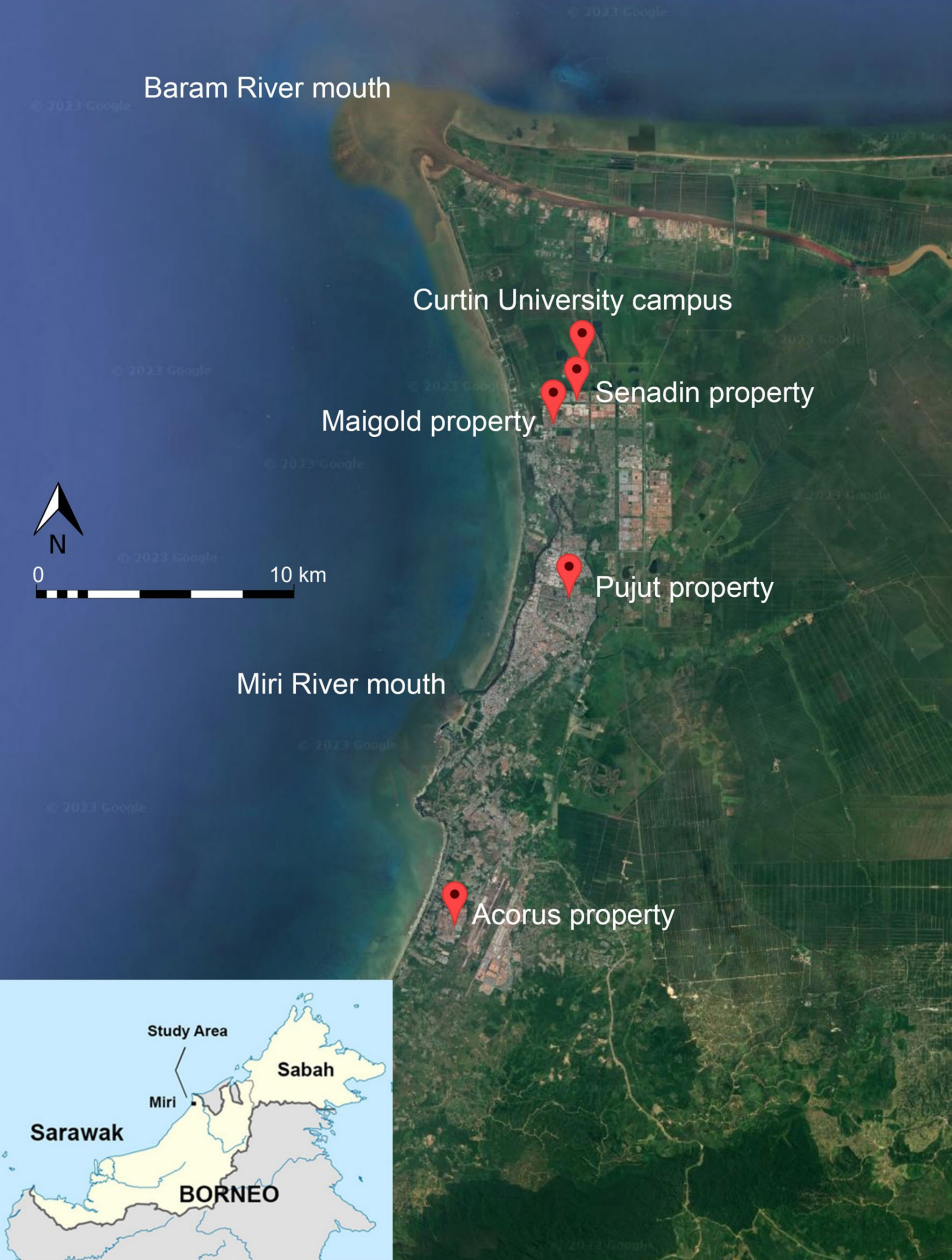
Map of study area showing location of the four properties (Senadin, Maigold, Pujut and Acorus) where micro-mapping of TGRD was carried out in Miri (Sarawak, Malaysia). The study area latitude ranges from 4.5028 in north (Senadin) to 4.3205 in south (Acorus) and from 113.9770 in west (Acorus) to 114.0166 in east (Senadin). Base image modified from Google Earth 2023

The research involves four properties in the urban area of Miri, a city in northern Sarawak which had a population of over 350,000 in 2020 [27]. Micro-mapping was carried out inside and outside of four properties in Miri, located as shown in Fig. 1. The properties are named here after the neighbourhood in which they are located or the adjacent street name: Pujut, Senadin, Maigold and Acorus (in order of decreasing number of measurements). The properties are spread out over a distance of approximately 20 km in a north–south direction covering most of Miri city. The age of the properties ranges from approximately 60 years (Pujut), to 14 years (Maigold and Acorus) and approximately 11 years (Senadin). The Pujut house is double-storey detached, whereas the other three properties are single-storey semi-detached. It should be noted that most urban homes in Malaysia, including these four properties, have tiled floor surfaces throughout. None of the properties have basements, and kitchens and washrooms are fitted with extractor fans and/or louvered windows to improve ventilation.

The city of Miri is built on a basement of sedimentary rocks of Middle Miocene age belonging to the Miri Formation with overlying Quaternary alluvium [28]. Miri Formation rocks consist of sandstones, mudstones and shales. Previous TGRD mapping has been carried out at Curtin University campus in the northern part of Miri city which is considered a greenfield site of equivalent underlying geology to that present at the four properties covered in this study [10]. The natural background TGRD average, away from campus buildings, was found to be 72 nGy/h. This is lower than the average across the whole of Malaysia (92 nGy/h) and lower than the average in the urban area of Kuala Lumpur [10].

## Methodology

Portable Polimaster PM1405 survey meters were used for both gamma and beta measurements. These instruments measure gamma and beta radiation using a Geiger-Muller counter in which radiation is transformed into electropulses [29]. The instruments are calibrated by the supplier’s Quality Control Department and considered valid for operation prior to use, to reduce instrumental error.

For Terrestrial Gamma Radiation Dose (TGRD) measurements, the instrument was positioned on a tripod one meter above ground level and allowed to stabilize until the statistical error percentage dropped below 10%. The readings of external environmental gamma radiation dose are expressed in sievert (Sv) the SI unit, and the instrument range is from 0.01 µSv/h to 130 mSv/h. A 1000 conversion factor was used to convert the readings in µSv/h to TGRD in nGy/h [10]. TGRD is the in situ measurement that is equivalent to air absorbed dose rates that can alternatively be determined from calculated activity concentrations of ^226^Ra, ^232^Th and ^40^K [17, 30]. For gamma, the instrument has measurement range 0.1 µSv/h to 100 mSv/h [29]. The limitation of this method is that the instrument is not significantly affected by potential presence of radon gas, for which other methods (such as passive devices left in place over several months) are commonly used [18, 21].

For beta measurements, the instruments were placed directly on the surfaces and two readings were taken. The first reading is joint beta plus gamma value (β + ϒ, also called beta flux) with the instrument screen filter in open position and after stabilization to less than 10% statistical error which may take several hours. The results are expressed in counts per second (CPS). The second reading is performed after saving the β + ϒ value, closing the instrument screen filter and again allowing stabilization, to measure the beta value alone in CPM/cm^2^ after subtraction of the background gamma signal [29]. For beta flux measurement the instrument range is 6.0 to 10 ^3^ CPM cm^−1^ [29].

Errors of observation were minimized by using standard method for all readings and all operators and allowing the instruments to stabilize to below 10% statistical error percentage.

The dimensions of rooms in the residences were measured using a Leica Disto D810 device and/or tape measure. The aim was to establish a 1 m by 1 m grid and acquire TGRD measurement data for every square meter within each residence, with additional measurements closer to walls in some areas. Additional data was also collected outside each residence to measure the background TGRD where no building materials are close or only present in part, such as on an open tiled patio or concrete surfaced parking space.

The annual effective dose equivalent (AED) was calculated following the method detailed in the literature as cited by Dodge-Wan and Mohan Viswanathan [10]. It is an estimate of the annual dose, resulting from both natural TGRD background outdoors for 20% of the time and TGRD indoors for 80% of the time.

Excess lifetime cancer risk has also been calculated, following the procedure stated in this paper.

## Results

**Fig. 2:**
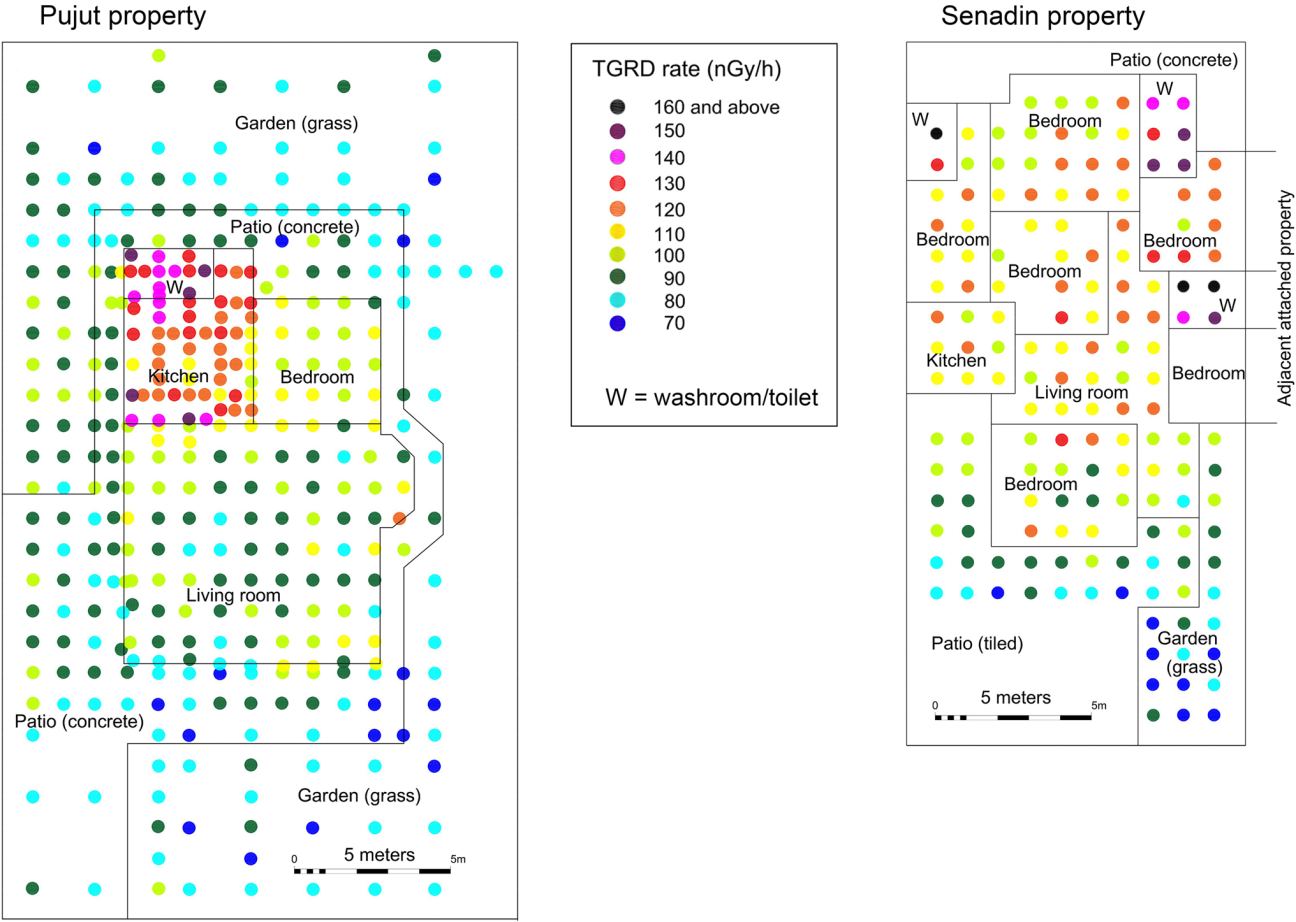
Micro-mapping of TGRD rate using 1 m by 1 m grid in and around two urban homes in Miri City: at Pujut (detached house) and at Senadin (semi-detached house). W denotes washroom or toilet

A total of 577 TGRD rate measurements were made in the four homes, and the results of this micro-mapping at Pujut and Senadin, where the highest number of readings were recorded, are shown in Fig. 2. At each property, the observed TGRD rate values have been grouped in the following four general categories according to the type of ground and wall covering materials:

- ON: Outdoors with natural surfaces (grass, soil) away from building walls or other man-made structures
- OM: Outdoors with mixed surfaces (for example concrete drive, patio, drain)
- I: Indoors in room with floor tiles (for example living room, dining room, bedroom, which typically have tiled floors)
- IWT: Indoors in room with floor tiles and wall tiles (for example washroom, kitchen and similar)

The TGRD values show a consistent minimum of 70 nGy/h within the four urban homes, with the lowest values recorded outdoors on natural surfaces, such as grass or soil. The maximum TGRD is 200 nGy/h in Maigold home, in a 2 m by 2 m sized washroom with grey floor tiles and yellow wall tiles. Slightly lower maximum TGRD value of 180 nGy/h was recorded at Senadin in a washroom of similar size, with textured black floor tiles and white wall tiles. The maximum TGRD at both Pujut and Acorus homes was 150 nGy/h, and was also recorded in washrooms.

**Fig. 3:**
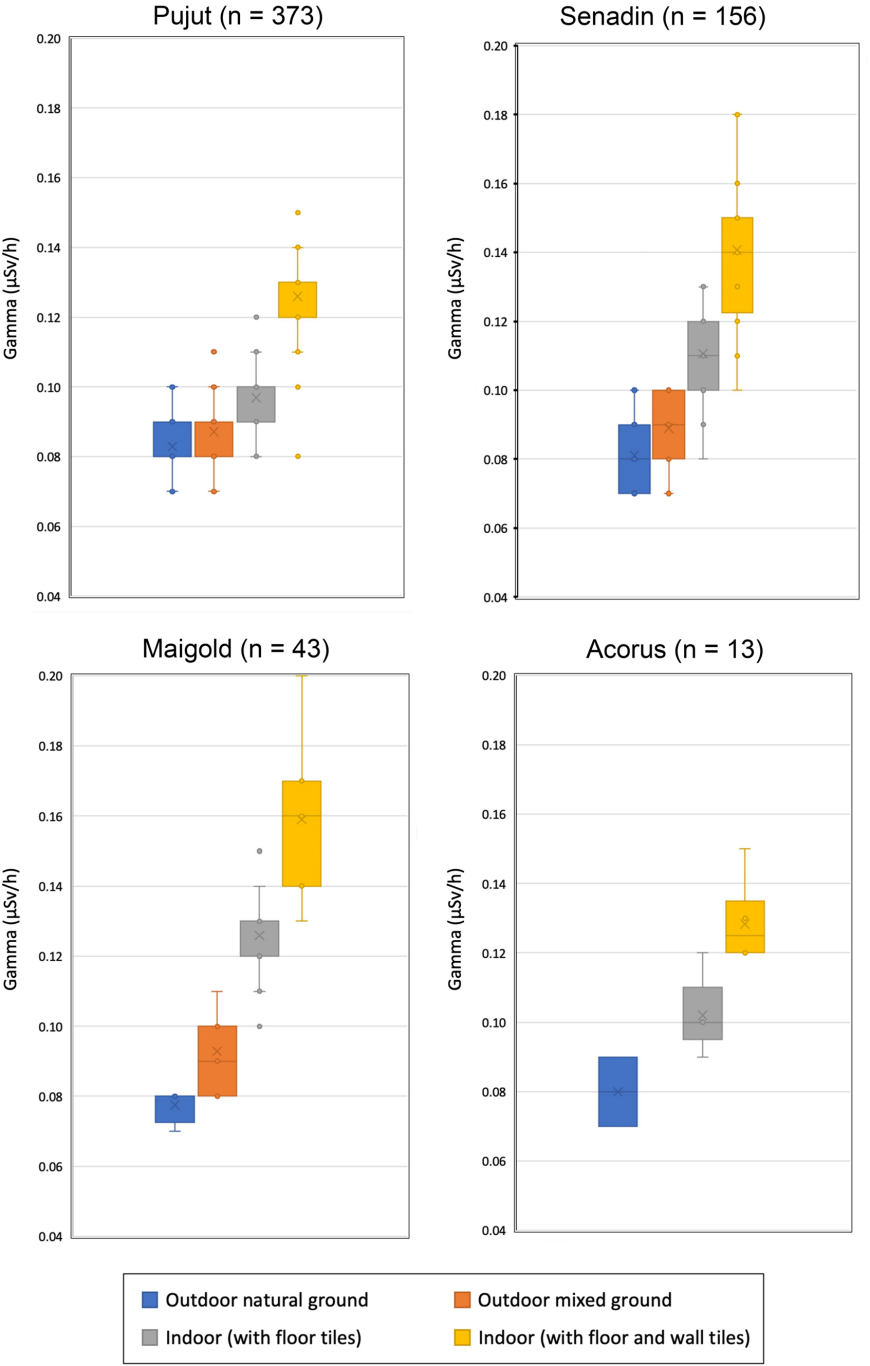
Box and whisker plots of gamma values in different environments in Pujut, Senadin, Maigold and Acorus homes, showing increase from outdoors on natural ground, to outdoors on mixed ground, to indoors with floor tiles to indoors with floor and wall tiles. The number of measurements (n) is given for each property

At all four homes, the TGRD is lowest in ON and OM categories and highest in I and IWT categories respectively as shown on Fig. 3 and in Table 1. The TGRD values were on average 5 to 19% higher outside on mixed surfaces, such as paved patio, compared to outside on natural surfaces. The TGRD were on average 17, 28, 36 and 62% higher inside the homes in rooms with tiled floors than compared to outside the homes on natural surfaces, at Pujut, Acorus, Senadin and Maigold properties respectively.

**Table 1:** Comparison of average, maximum and minimum TGRD across sites and different environments

Total number of measurements (n)		Urban homes in Miri City				
		Pujut	Senadin	Maigold	Acorus	Curtin University Campus [10]
		365	156	43	13	143
*Average TGRD in nGy/h*						
ON: Outdoors (natural surfaces)		83	81	78	80	77
OM: Outdoors (mixed surfaces)		87	89	93	–	85
I: Indoors (floor tiles only)		97	110	126	102	110
IWT: Indoors (floor and wall tiles)		125	118	159	128	135
Maximum TGRD in nGy/h		150	180	200	150	150
Minimum TGRD in nGy/h		70	70	70	70	50
ON to OM increase		5%	10%	19%	–	10%
ON to I increase		17%	36%	62%	28%	43%
I to IWT increase		29%	7%	26%	25%	23%
ON to IWT increase		51%	46%	103%	60%	75%
Indoor (I) to outdoor (ON) ratio		1.17	1.36	1.62	1.28	1.43

The TGRD were on average 51 to 103% higher in rooms where both floor and walls are tiled than compared to outside the homes on natural surfaces. When comparing TGRD in rooms with only tiled floors and in rooms with both tiled floors and tiled walls, across all four homes the increase ranges from 7 to 29%.

**Fig. 4:**
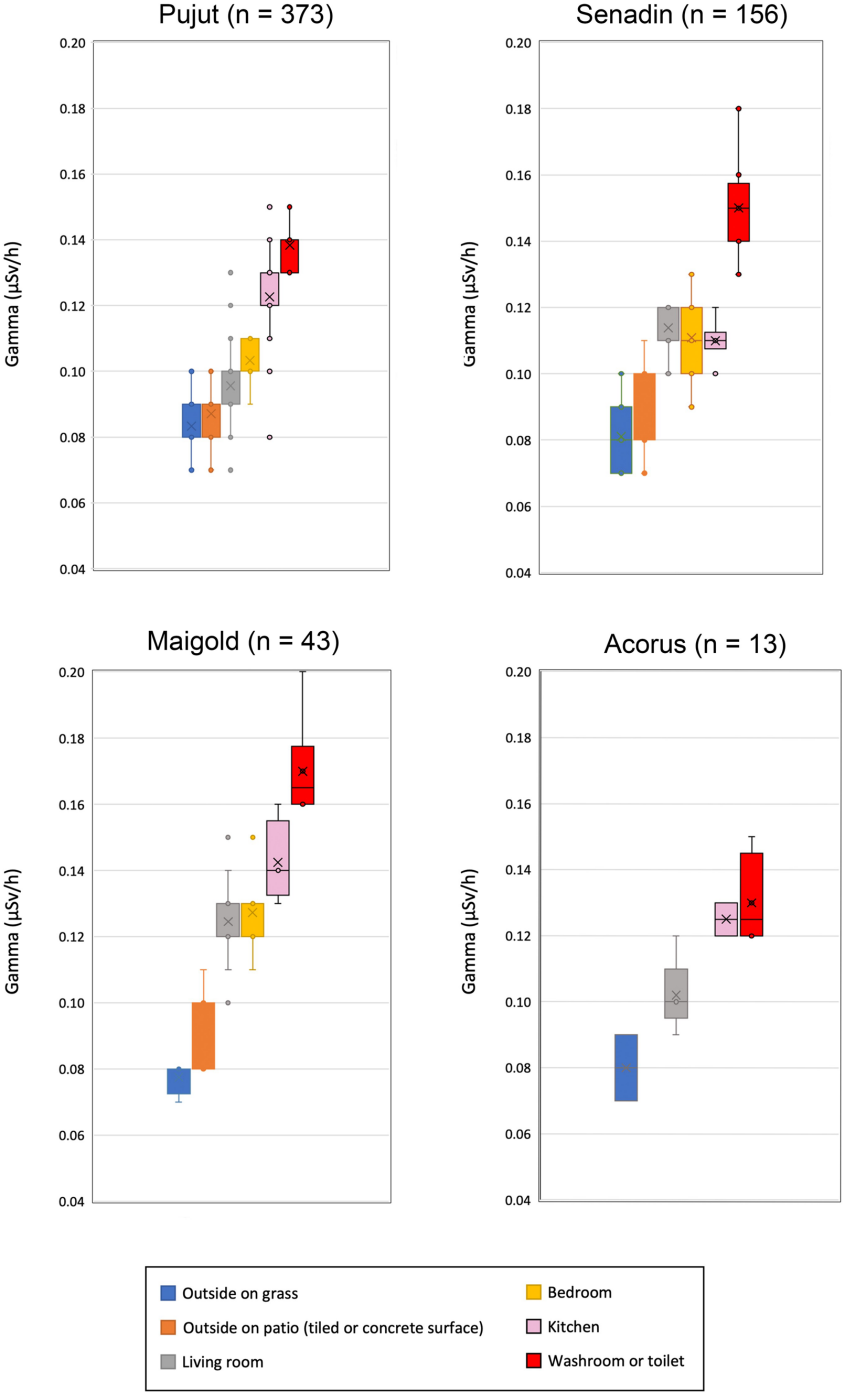
Box and whisker plots of gamma values in different spaces and room types in Pujut, Senadin, Maigold and Acorus homes, showing highest values in rooms with tiled walls such as kitchens, washrooms and toilets. The number of measurements (n) is given for each property

In order to better understand the variation of TGRD rates within the homes, the measurements were grouped according to the type of room, or living space as follows: outside on grass, outside on patio with tiled or concrete surface, indoors in living room (includes halls and dining areas), indoors in bedroom, indoors in kitchen and indoors in washroom and toilet (Fig. 2). All of the indoor rooms in all of the properties have tiled floors. The kitchens also have tiled walls (Pujut, Maigold and Acorus) or partially tiled walls (Senadin), whereas the washrooms and toilets have fully tiled walls. Table 2 provides the average TGRD values according to these room types. At all four properties a clear step-wise increase in TGRD is noted from outside on grass, outside on patio, to inside in room with tiled floor and further increasing in rooms with floor and wall tiles (kitchens and washrooms) as illustrated in the box and whisker plots of Fig. 4. The maximum and highest room average TGRD values at each property were consistently found in the washrooms and toilets. In washrooms and toilets, the average TGRD values were 63 to 118% higher than outside on grass.

**Table 2:** Comparison of average TGRD according to room type in four urban homes in Miri

	Average TGRD in nGy/h			
	Pujut	Senadin	Maigold	Acorus
Outside garden on grass	83	81	78	80
Outside on tiles or concrete patio	87	91	93	–
Living room with tiled floor	96	114	125	100
Bedroom with tiled floor	103	111	127	–
Kitchen with tiled floor and walls	123	110	143	125
Washroom or toilet with tiled floor and walls	138	150	170	130

There are considerable differences in TGRD values indoors in living room, bedroom kitchen and washrooms, between the homes at Pujut, Senadin and Maigold, the three homes for which a large amount of data on TGRD was measured. Table 2 and Fig. 4 show that across all the rooms, the Pujut property has the lowest TGRD values except for in the kitchen. On the other hand, the Maigold property has the highest TGRD values. The relatively low value recorded in the kitchen at Senadin might be due to the fact that Senadin kitchen has walls that are only partially tiled, whereas at Pujut and Maigold the kitchens are fully wall-tiled. The differences between TGRD values in specific rooms across the properties range from 23% (bedrooms, kitchens, washrooms) to 30% (living rooms).

**Fig. 5:**
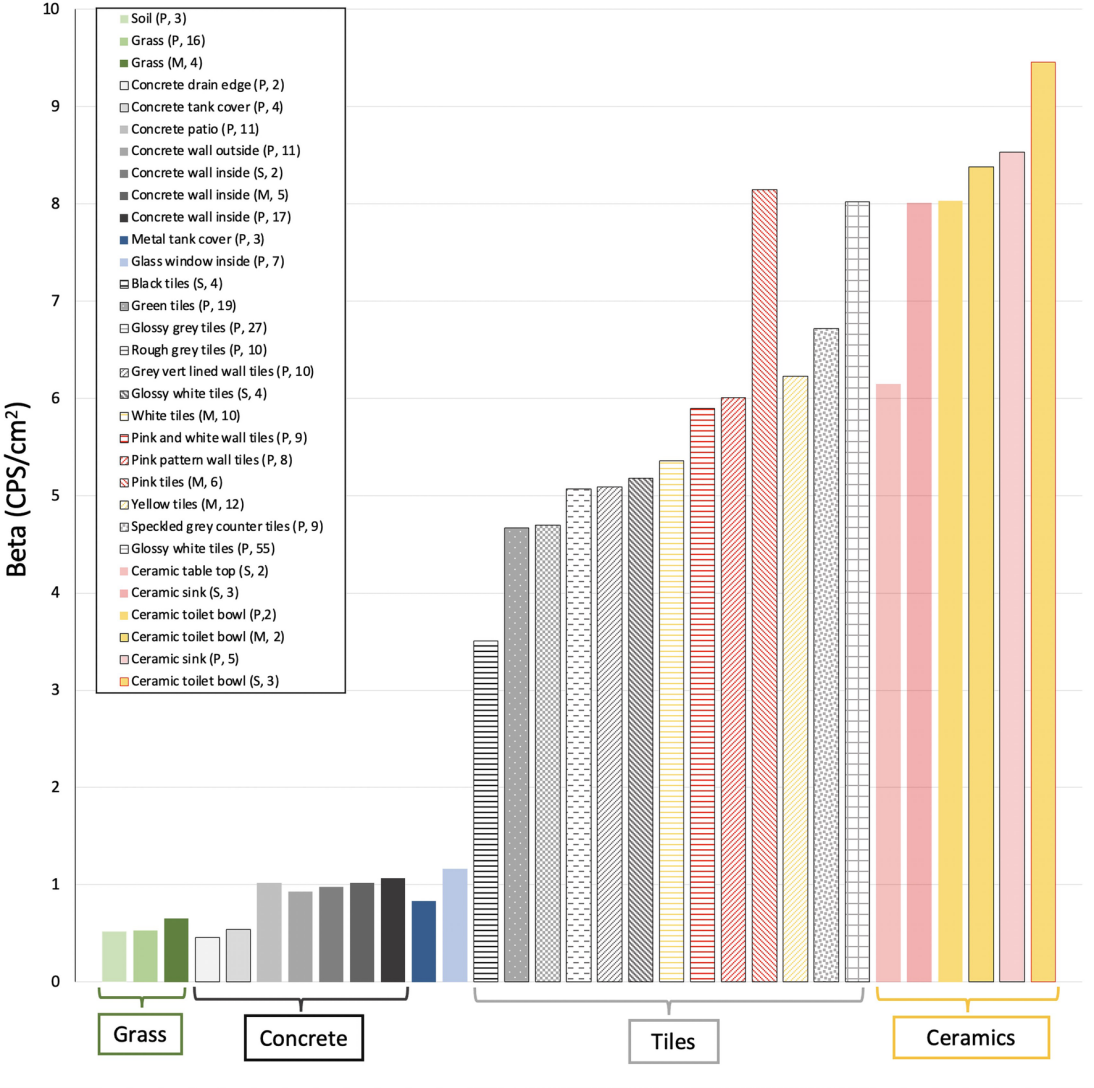
Average beta values in CPS/cm^2^ recorded on variety of surfaces of different construction materials in the Pujut (P), Senadin (S) and Maigold (M) homes. The numbers in brackets in the legend indicates the number of beta measurements for each type of surface in the respective homes indicated by letter

**Fig. 6:**
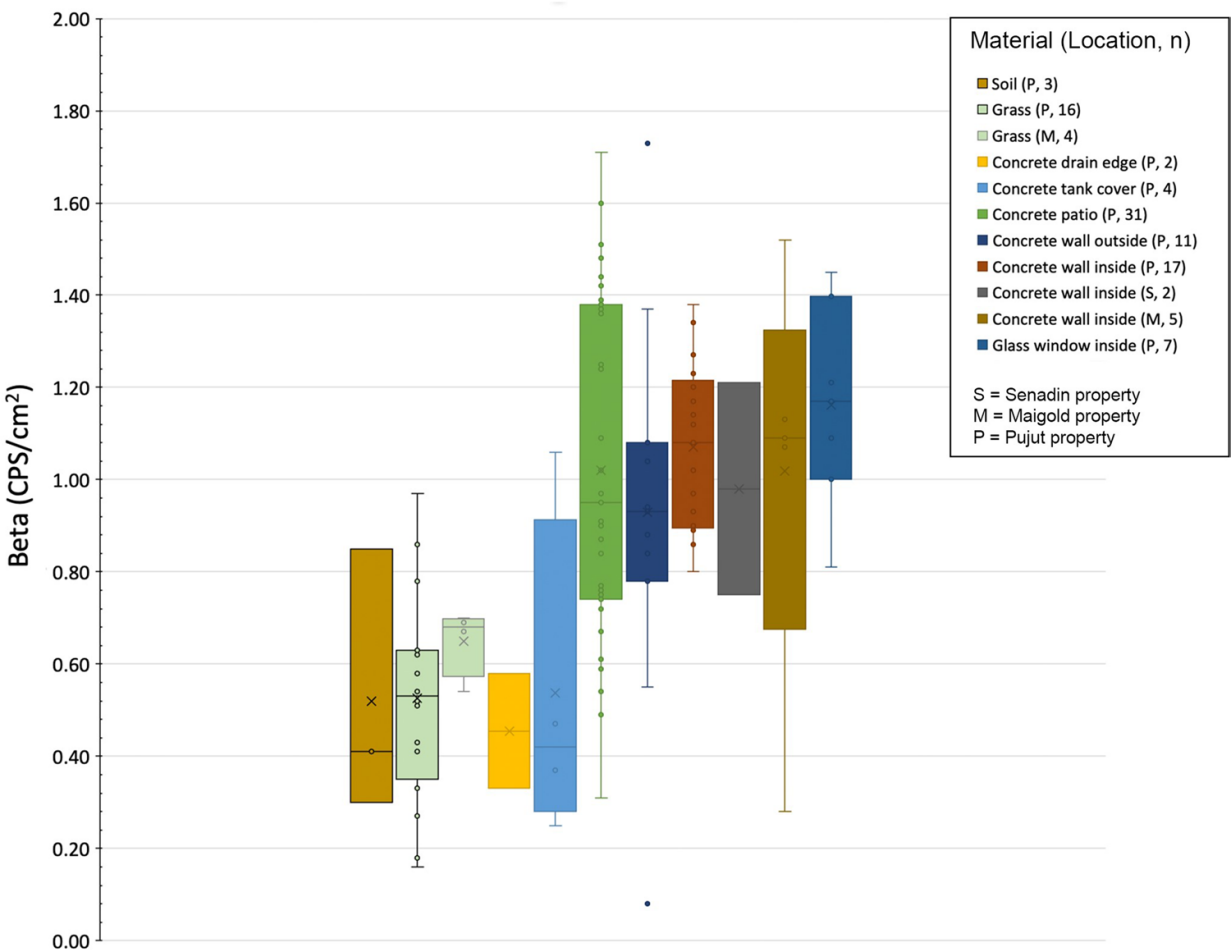
Consistency of beta radiation values on non-tiled surfaces at the different properties examined, showing the clustering of low values for soil, grass and concrete all below 1.8 CPS/cm^2^. Letters in brackets in legend denote the home, and numbers denote the number of measurements for each type of surface. Note: the vertical axis scale of this figure differs from that shown in Fig. 7

**Fig. 7:**
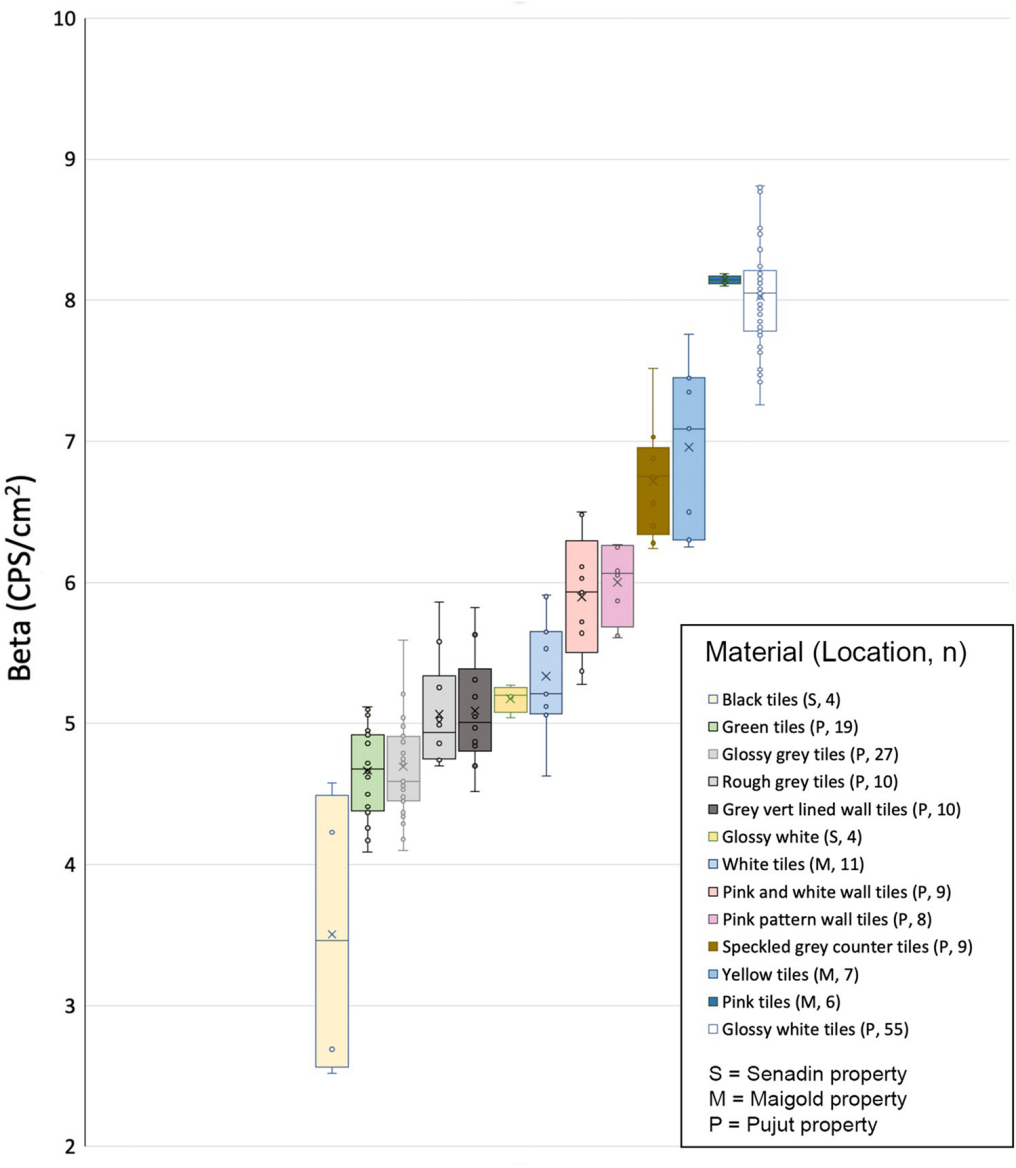
Variability of beta radiation values on the 13 different types of tiles examined in Pujut, Senadin and Maigold homes in Miri. Yellow bars are for Senadin (S) property, blue bars for Maigold (M) property and the remainder are from Pujut (P) home. Letters in brackets in legend denote the home, and numbers denote the number of measurements for each type of tile

A variety of construction materials are present in the homes: concrete surfaces, walls, glass windows, floor tiles, wall tiles and ceramic bathroom fixtures. In each home, a number of beta radiation values (in CPS/cm^2^) were measured on each type of surface, including measurements on each different type of tile present in each of the properties. In all, over 300 beta values were measured. The results are summarized in Table 3 and shown in Figs. 5, 6 and 7.

**Table 3:** Summary of beta results on different building materials in four urban homes in Miri City

		Natural surfaces (soil and grass)	Concrete surfaces (patio, walls etc.)	Tiled surfaces (floor and wall tiles)	Ceramic bathroom and kitchen fixtures (sink, toilet bowl, table top)
Total number of measurements		23	72	183	10
Beta values in CPS/cm^2^	Maximum	0.97	1.73	8.81	13.50
	Minimum	0.16	0.08	2.52	4.72
	Average	0.55	0.98	6.18	8.15

The results for natural surfaces such as soil and grass were found to be low and consistent between properties as shown on Fig. 6, with an overall average beta value of 0.55 CPS/cm^2^ (23 measurements). Concrete surfaces, including patio surfaces, drain edges, septic tank covers, interior and exterior walls were also found to be consistent with a slightly higher average beta of 0.98 CPS/cm^2^ (72 measurements).

A total of 13 types of tiles were present in the properties and 183 measurements indicate an overall average for tiles of 6.19 CPS/cm^2^. In all the properties, the beta values for tiles were found to be systematically higher than for the natural or concrete surfaces (Fig. 5) and variable. The minimum reading recorded on tiles was 2.52 CPS/cm^2^ (textured black tiles at Senadin) and the maximum was 8.81 CPS/cm^2^ (glossy white floor tiles at Pujut). Significant differences were found between the average beta for different types of tiles, as shown in Fig. 7. The lowest average beta was 3.51 CPS/cm^2^ on black tiles at Senadin home. Eight of the 13 tile types had average beta values between 4 and 6 CPS/cm^2^. Four of the types had average beta values in 6 to 9 CPS/cm^2^ range, with the highest average being 8.15 CPS/cm^2^ (pink tiles at Maigold).

## Calculated annual effective dose

To compare the amount of radiation a person receives from their surroundings in a year with established limits and standards, it is common practice to calculate the annual effective dose (AED). The formula provides AED in mSv, based on the assumption that an individual spends 80% of their time indoors and 20% of their time outdoors in a year (8760 h) [10]. For application of the formula, TGRD values in nGy/h are required for both indoor and outdoor environments. A coefficient of conversion of 0.7 adapted by UNSCEAR is used to convert absorbed dose rate in air to effective dose in adult humans giving the following formula [10]:

1 $${\text{AED}}\left( {{\text{in}}\;{\text{mSv}}} \right) \, = \, \left( {{\text{outdoor}}\;{\text{TGRD}} \times {2}0\% + {\text{indoor}}\;{\text{TGRD}} \times {8}0\% } \right) \times {876}0 \times 0.{7} \times {1}0^{{ - {6}}}$$

which can be expressed as:

2 $${\text{AED}}\left( {{\text{in}}\;{\text{mSv}}} \right) = \left( {{\text{D}}_{{{\text{out}}}} \times {2}0\% + {\text{D}}_{{{\text{in}}}} \times {8}0\% } \right) \times {876}0 \times 0.{7} \times {1}0^{{ - {6}}}$$

It is common practice to apply formula (2) using a single D_out_ value for outdoor TGRD and another single D_in_ value for indoor TGRD, irrespective of how these values were obtained [1, 31–33]. In this study, which generated a large amount of actual measured in situ data on both indoor and outdoor gamma dose rates in specific homes and in the specific inhabitable spaces within those homes, we propose to apply a more detailed and novel method to assess AED. The proposed method is based on formula (1) but with specific outdoors and indoor TGRD values (D_out_ and D_in_) based on the findings of micro-mapping for each inhabited space i.e. type of room and in each home.

Table 4 outlines four scenarios that were considered here in the calculation of AED using the formula. In the scenario 1 a single D_out_ value for outdoors on natural ground was applied for 20% of the time and a single D_in_ value for average in a room with only floor tiles (such as typical living room) was applied for the remaining 80% of time. Scenario 1 method can be considered as the standard calculation as applied in most studies, and where there is limited data (single value for D_out_ and D_in_) [1, 31–33]. The scenario 2 is more detailed in that the specific TGRD values obtained by micro-mapping each type of inhabited space in each home are applied. For time spent outdoors, it was subdivided into 10% time on grass and 10% time spent on tiled or concreted patio. For time spent indoors, it was subdivided into 35% time spent in living room (for example 8.4 h for a person working from home), 35% time spent in bedroom (for example 8.4 h typical sleeping or in bedroom), 5% time spent in kitchen (1.4 h) and 5% time spent in washroom or toilet (1.4 h). The later two spaces may typically have tiled walls in urban homes. Scenario 2 represents the closest estimate to the actual realistic situation for calculation of AED where a lot of data is available. The scenario 3 is based on a fictitious home in which the single highest average TGRD for each type of inhabited space was used, based on the results of this micro-mapping study in four urban homes. Scenario 3 assumes that the highest average values observed anywhere in this study were all present together in a single home and applied in each space of that fictitious home. For scenario 4, it was considered that in addition to this, the inhabitant spent a larger proportion of their time in the specific spaces that have the higher TGRD values (such as 3.6 h spent in washrooms and toilets). Scenario 4 represents a fictitious “worst-case scenario”, and is unlikely to be exceeded in homes of this type in Miri.

**Table 4:** Detail of scenarios applied in the use of AED formula to better assess AED in properties where large amount of data is available for various inhabited spaces of the homes

Scenario number	Outdoor TGRD values used (D_out_)	Indoor TGRD values used (D_in_)
Scenario 1 (single D_out_ and D_in_ values)	20% time: average value for outdoors on natural ground (ON)	80% time: average value for indoors in rooms with floor tiles only (I), used living room values
Scenario 2 (specific D_out_ and D_in_ values for each inhabited space in each home)	10% time: average value for outdoors on natural ground (ON), for example on grass 10% time: average value for outdoors on mixed ground (OM), for example on tiled or cemented patio	35% time: average for living room 35% time: average for bedroom 5% time: average for kitchen 5% time: average for washrooms and toilets
Scenario 3 (highest values applied in each inhabited space)	10% time: highest average value for outdoors on natural ground (ON), for example on grass 10% time: highest average value for outdoors on mixed ground (OM), for example on tiled or cemented patio	35% time: highest average for living room 35% time: highest average for bedroom 5% time: highest average for kitchen 5% time: highest average for washrooms and toilets
Scenario 4 (highest D_out_ and D_in_ values applied for each inhabited space and more time spent in spaces with highest values)	20% of time spent outdoors on mixed ground (OM), for example on tiles or cemented patio	5% time: use highest average for living room 35% time: use highest average for bedroom 25% time: use highest average for kitchen 15% time: use highest average for washrooms and toilets

The results of AED calculations using the four scenarios are given in Table 5. The results for scenario 1 indicate that the AED ranges from 0.573 mSv at Pujut to 0.709 mSv at Maigold, which is a 24% difference between properties. The results for more realistic scenario 2, using large number of in situ TGRD measurements on the specific characteristics of each home and their respective building materials, confirm that there is a considerable difference in the annual exposure dose for inhabitants of the different homes. The Pujut home, which is the oldest property, had the lowest AED of 0.611 mSv and Maigold, one of the more recent properties, had AED of 0.742 mSv which is 21% higher. The other properties were 5% (Acorus) and 9% (Senadin) higher AED compared to Pujut.

**Table 5:** Calculated AED values using four different scenarios

	Calculated parameters:	Type of space	Pujut	Senadin	Maigold	Acorus
Scenario 1	D_out_ in nGy/h (% time)	Outdoors on grass	83 (20%)	81 (20%)	78 (20%)	80 (20%)
	D_in_ in nGy/h (% time)	Indoors in living room	96 (80%)	114 (80%)	125 (80%)	100 (80%)
	AE _out_ in mSv	–	0.102	0.099	0.096	0.098
	AE _in_ in mSv	–	0.476	0.540	0.618	0.500
	Calculated AED in mSv	–	0.573	0.659	0.709	0.598
Scenario 2	D_out_ in nGy/h (% time)	Outdoors on grass Outdoors on patio	83 (10%) 87 (10%)	81 (10%) 91 (10%)	78 (10%) 93 (10%)	80 (10%) 90 (10%)
	D_in_ in nGy/h (% time)	Living room Bedroom Kitchen Washroom and toilet	96 (35%) 103 (35%) 123 (5%) 138 (5%)	114 (35%) 111 (35%) 110 (5%) 150 (5%)	125 (35%) 127 (35%) 143 (5%) 170 (5%)	100 (35%) 114 (35%) 125 (5%) 130 (5%)
	AE_out_ in mSv	–	0.104	0.105	0.105	0.104
	AE_in_ in mSv	–	0.507	0.563	0.537	0.537
	Calculated AED in mSv	–	0.611	0.668	0.742	0.642

	Calculated parameters:	Type of space	Fictitious property
Scenario 3	D_out_ in nGy/h (% time)	Outdoors on grass Outdoors on patio	83 (10%) 93 (10%)
	D_in_ in nGy/h (% time)	Living room Bedroom Kitchen Washroom and toilet	125 (35%) 127 (35%) 143 (5%) 170 (5%)
	AE_out_ in mSv	–	0.108
	AE_in_ in mSv	–	0.637
	Calculated AED in mSv	–	0.745
Scenario 4	D_out_ in nGy/h (% time)	Outdoors on patio	93 (20%)
	D_in_ in nGy/h (% time)	Living room Bedroom Kitchen Washroom and toilet	125 (5%) 127 (35%) 143 (25%) 170 (15%)
	AE_out_ in mSv	–	0.114
	AE_in_ in mSv	–	0.686
	Calculated AED in mSv	–	0.801

Assuming a property with the highest observed TGRD for each space, i.e. a property which combines all the high averages for the respective building materials in one home, could lead to AED of 0.755 mSv (scenario 3), which is 22% higher than Pujut and similar to the Maigold home where highest TGRD values were actually observed. The calculation results for scenario 4, in which a person spends a lot of time in the rooms with highest TGRD, show it would be possible to reach AED of 0.801 mSv in this worst-case scenario. This is 31% higher than the realistic scenario 2 at Pujut with the difference being in the specific TGRD of the rooms and the amount of time spent in them.

## Excess lifetime cancer risk

Excess lifetime cancer risk (ELCR) is a calculated indication of the additional risk that a person would develop cancer due to exposure to cancer-causing substances, over and above the “normal” risk without exposure to those substances. It is “the difference between the proportion of people who develop or die from the disease in an exposed population and the corresponding proportion in a similar population without the exposure” [34].

Excess lifetime cancer risk (ELCR) is calculated as follows:

3 $${\text{ELCR}} = {\text{AED}} \times {\text{DL}} \times {\text{RF}}$$

With AED being expressed in Sv/y, DL being the duration of life taken as 70 years, and RF being the fatal cancer risk factor for which ICRP adopts the value of 0.055 Sv ^−1^ for the public [34].

It has been established that ELCR values of 0.001, 0.01, 0.1 and 1 Sv respectively result in an increase in the chance of developing fatal cancer of 0.004, 0.04. 0.4 and 4 per cent [35]. For this study, the calculated ELCR under the scenarios presented for the calculation of AED are given in Table 6 for comparison with data from Curtin University campus in Miri [10], Malaysian and world averages [1]. The results obtained by this study suggest that using single value for D_out_ and D_in_ may lead to an underestimation of the ELCR (scenario 1) compared to more realistic calculation that considers the specific TGRD in each room and typical time spent in them (scenario 2), with differences of the order of 0.2 × 10^–3^ in ELCR. The results of micro-mapping indicate that in worst-case scenario (scenario 4) in properties of this type, the ELCR might be up to 0.8 × 10^–3^ above the underestimated value obtained with the standard calculation (formula (2), scenario 1).

**Table 6:** Calculated ELCR under various scenarios and for the four properties in Miri City, compared to Curtin Campus in Miri, Malaysian and world averages. The Malaysian average used D_out_ 92 nGy/h and D_in_ 96 nGy/h and the world average used D_out_ 59 nGy/h and D_in_ 84 nGy/h, based on data from UNSCEAR [1]. The Curtin Campus value used D_out_ 82 nGy/h and D_in_ 112 nGy/h [10]. Scenarios are described in Table 4

	Excess Lifetime Cancer Risk (ELCR) × 10^–3^						
	Pujut	Senadin	Maigold	Acorus	Curtin Campus [10]	Malaysian average [1]	World average [1]
Scenario 1	2.005	2.305	2.481	2.060	2.264	2.043	1.695
Scenario 2	2.104	2.338	2.596	2.246	–	–	–
Scenario 3	2.607				–	–	–
Scenario 4	2.802				–	–	–

## Discussion

The minimum TGRD and averages for outdoors (natural surfaces) are slightly higher in the urban areas (i.e. the gardens of the four homes) than those reported on Curtin University campus built on a greenfield site near Miri [10]. This suggests that the TGRD might still be influenced by building materials to some distance, estimated at a few meters away from those materials, as for example in gardens close to properties where there may be walls, covered patios and other materials. The maximum TGRD are consistent across all the properties and also Curtin University site [10] – they are also consistently highest in small rooms with tiles floors and walls i.e. in typical washrooms.

The outdoor average TGRD values obtained in this study, given in Table 1, are all below the reported Malaysian average [1]. As mentioned, the geology of the area is not expected to have high background radiation, being essentially quartz-rich sedimentary rocks.

This study indicates indoor-to-outdoor ratios for TGRD that range from 1.17 to 1.62 with the lowest ratios in the oldest property (Pujut) and higher ratios in new properties. The ratio at Curtin University campus in Miri was reported to be 1.43 which is within this range [10]. UNSCEAR [1] report Malaysian average of 92 nGy/h outdoors and 96 nGy/h indoors, so a ratio of 1.04. More TGRD data has been obtained by a number of authors since 2000 and has been summarized by Dodge-Wan and Mohan Viswanathan [10] which indicates significant variability outdoors in several areas of Malaysia, with some outdoor averages exceeding 200 or even 300 nGy/h in high radiation hot spots. UNSCEAR [1] indicates a world average indoor-to-outdoor ratio of 1.4.

The Malaysian average indoor TGRD is reported to be 96 nGy/h [1]. This study has obtained a very high number of readings, rarely obtained in other studies of indoor radiation. Previously Sulaiman and Omar [36] reported an average indoor TGRD value of 42 nGy/h for 20 towns in Sarawak state. This is significantly lower than the averages obtained in this study which range from 97 to 159 nGy/h depending on the rooms. It is thought that the difference may be due to the fact that Sulaiman and Oman studied a range of houses made of concrete, brick and wood including wooden houses in water villages [36]. This study targeted the concrete constructions with tiled floors i.e. the urban homes in areas built up over the last 60 years.

Although there is a research gap on micro-mapping of TGRD inside buildings, a number of studies have measured gamma radiation in various dwellings around the world. With large difference in local geology that can be expected to affect the results, in addition to differences in building styles, materials and other factors, it is not appropriate to directly compare with the results of this study in four urban homes in Miri. There are however, a number of findings are relevant.

Miah [19] noted and inverse linear relationship with correlation coefficient of -0.96 between building age and annual average dose rate in 15 houses around the Atomic Energy Research Establishment at Savar, Bangladesh and suggests that this relationship might be due to various factors including the materials used in the constructions. In Miri, it is noted that the oldest property at Pujut showed the lowest TGRD and AED values, with higher values in newer properties at Maigold and Senadin. However, it is not known if this is due to a change in the type of building materials used or other factors. Al-Ghorabie [20] found indoor gamma dose rates were highest in apartments and villas, compared to large halls and mud houses with average values of 192, 154, 167, 92 nGy/h respectively with some of the difference attributed to the building materials and some to the degree of ventilation, as well as the season. The values recorded in Miri are comparable to those of villas. Al-Saleh [22] noted highest AED in bathrooms in Riyadh city, Saudi Arabia, with values of 0.423 to 0.700 mSv and some variation between different sectors of the city. This study in Miri also found highest values in the bathrooms.

Numerous studies have shown that commercial tiles, frequently contain zircon which is used for glazing, and the presence of zircon can lead to higher concentrations of naturally occurring radionuclides [9, 17]. Whilst the results of this study, using hand-held sensors, so cannot be directly compared to studies based on measured activity concentrations of the radionuclides, they do clearly show that the presence of tiles in typical urban homes increases the gamma radiation. This leads to higher TGRD rates in kitchen, washrooms and toilets which typically have tiled walls in addition to the tiled floors that are found throughout all rooms in most urban homes in Malaysia. Higher gamma radiation leads to higher AED and this study showed 28% difference between typical properties. In the worst-case scenario of a person spending a lot of time in rooms with the highest TGRD, this could lead to a 33% increase in AED. This study also shows that tiles and ceramics have higher beta radiation of 6 to 8 times that recorded on concrete, with variability between different tile types. It would be advantageous, in future studies, to measure both the values in situ, as done in this study, together with measuring the activity concentration of radionuclides in the specific building materials found in these homes.

Micro-mapping has shown that in typical urban homes in Miri, there is considerable variation in TGRD values within each home according to the presence of different building materials. The use of single D_out_ and D_in_ values in the calculations (as in scenario 1) may lead to an underestimate of AED and ELCR. The results shown in Table 5 suggest that the underestimate (between scenario 1 and more realistic scenario 2) is of the order of 1% to 7%.

**Fig. 8:**
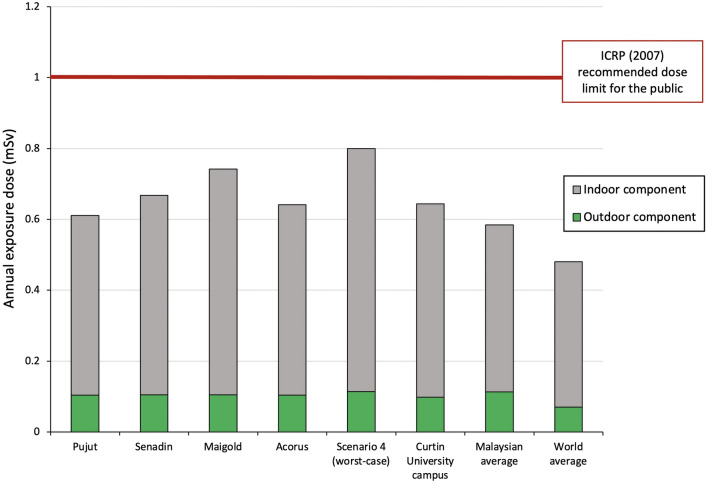
Annual exposure dose measured in situ at four properties in Miri City (Pujut, Senadin, Maigold and Acorus) compared to worst-case calculated value for similar properties in Miri and published values for Curtin University campus [10], for Malaysia and world [1]. ICRP (2007) limit is from [34]

Figure 8 shows the indoor and outdoor components of AED in the four properties, in the worst-case scenario in comparison to results from Curtin University campus in Miri [10] and those reported for Malaysia and worldwide [1]. There is very little variation in the outdoor component of AED but approximately 33% variation in indoor component either measured or calculated for worst-case in Miri. The worst-case scenario estimate, based on a person spending a long period of time in rooms with the highest likely TGRD for this type of homes, amounted to AED of 0.801 mSv. The more realistic scenario calculations of annual effective dose based on the in situ measurements of this study range from 0.611 to 0.742 mSv. All values of AED for Miri fall below the ICRP [34] recommended effective dose limit of 1 mSv/y coming from all radiation sources, for public exposure although they are above the world average of 0.48 mSv.

## Conclusions

This micro-mapping study conducted during Covid-19 pandemic lockdown, obtained a very high number of indoor TGRD readings, rarely obtained in other studies of indoor radiation. The focus was four homes in typical urban areas of Miri, Sarawak. A total of 577 gamma and 300 beta readings were obtained in the various rooms of these homes which is a significantly large data set with which to assess the external exposure component of radiation impact of building materials in these specific homes.

The results indicate a clear step-wise increase in TGRD from a background of approximately 80 nGy/h outside on grass, slightly increasing outside on patio, increasing more inside in rooms with tiled floors and with the highest TGRD being recorded in rooms with both floor and wall tiles, such as kitchens and bathrooms. The indoor room averages range from 96 nGy/h (living room at Pujut) to 170 nGy/h (washroom at Maigold). Hence, the study revealed significant differences in the TGRD values between the homes and between the rooms in each home. On the whole, the oldest property has the lowest TGRD values, with higher values in more recent constructions, possibly reflecting differences in the use or source of various building materials. In addition, lower ventilation rates may play a role, allowing for accumulation of radon in some rooms, although all rooms are relatively well ventilated, with extractor fans and/or louvered windows common in kitchens and washrooms. Outdoor TGRD values were slightly higher adjacent to the urban homes than previously reported at greenfield site in Miri [10] but are below the Malaysian average [1]. It should be noted that the Malaysian average is based on data that was collected over 20 years ago [1]. In this study indoor-to-outdoor ratios of 1.17 to 1.62 were recorded.

Beta readings show significant differences between natural surfaces and tiles, with tiles having 8 to 15 times higher beta radiation than grass. Beta radiation was measured on 13 different types of tiles in use in the four homes. The values range from 2.52 CPS/cm^2^ to 8.81 CPS/cm^2^ with significant differences between the types.

Annual effective dose was calculated for a range of scenarios. In the studied homes, AED ranges from 0.611 mSv to 0.742 mSv. The numerous data obtained from micro-mapping have made it possible to calculate that in a worst-case scenario, a person living in a property of this sort might receive up to 0.801 mSv annual effective dose, but it is unlikely that the dose would be exceeded in properties of this type in this region. The value is below the 1 mSv dose limit for public recommended by the International Commission on Radiological Protection [34]. This study is significant in that it shows that having only limited data for indoors (for example only a single indoor value for each property) can lead to a potential underestimation of AED of the order of 30%. To minimize annual effective dose, it is recommended to use available building materials with the lowest radiological impact and this is most critical for tiles especially those typically used for flooring throughout Malaysian homes and for wall surfaces in washrooms and kitchens.
